# Patterns and Emerging Trends in Acute Poisoning with Substances of Abuse Used for Recreational Purposes in Adolescents: A Six-Year Multicentre Study

**DOI:** 10.3390/life14081033

**Published:** 2024-08-20

**Authors:** Teodora-Adela Turcu, Andreea Lescaie, Andreea Rodica Grama, Andreea-Cătălina Strătulă, Andreea-Iasmina Vincene, Laura-Maria Grigoraș, Cristina Jităreanu, Alina Maria Babeu, Mihai Gafencu, Maria-Dorina Crăciun, Carmen-Daniela Chivu, Daniela Luiza Baconi, Cristina Maria Mihai, Coriolan Emil Ulmeanu, Gabriela Viorela Nițescu

**Affiliations:** 1 Department of Pediatrics, “Carol Davila” University of Medicine and Pharmacy, 020021 Bucharest, Romania; teodora-adela.turcu@drd.umfcd.ro (T.-A.T.); rodica-andreea.grama@umfcd.ro (A.R.G.); andreea.stratula@stud.umfcd.ro (A.-C.S.); andreea-iasmina.vincene@rez.umfcd.ro (A.-I.V.); grigoras.lauramaria@gmail.com (L.-M.G.); coriolan.ulmeanu@umfcd.ro (C.E.U.); viorela.nitescu@umfcd.ro (G.V.N.); 2 Pediatric Poison Centre, “Grigore Alexandrescu” Clinical Emergency Hospital for Children, 017443 Bucharest, Romania; 3 Pediatric Poison Centre, “Saint Mary” Clinical Emergency Hospital for Children, 700309 Iași, Romania; cristina_jitareanu@yahoo.com; 4 Department of Pediatrics, Apollonia University, 700511 Iași, Romania; 5 Emergency Department, “Louis Turcanu” Clinical Emergency Hospital for Children, 300011 Timișoara, Romania; alinababeu@yahoo.com; 6 Pediatric Poison Centre, “Louis Turcanu” Clinical Emergency Hospital for Children, 300011 Timișoara, Romania; mgafencu@umft.ro; 7 Department of Pediatrics, “Victor Babeș” University of Medicine and Pharmacy, 300041 Timișoara, Romania; 8 Department of Epidemiology, “Carol Davila” University of Medicine and Pharmacy, 010221 Bucharest, Romania; maria.craciun@umfcd.ro (M.-D.C.); carmen-daniela.chivu@drd.umfcd.ro (C.-D.C.); 9 Department of Infection Prevention and Control, Grigore Alexandrescu Clinical Emergency Hospital for Children, 011743 Bucharest, Romania; 10 Department of Toxicology, “Carol Davila” University of Medicine and Pharmacy, 020021 Bucharest, Romania; daniela.baconi@umfcd.ro; 11 Department of Pediatrics, “Ovidius” University of Medicine and Pharmacy, 900470 Constanta, Romania; 12 Pediatric Poison Center, County Clinical Emergency Hospital of Constanta, 900470 Constanta, Romania

**Keywords:** substance abuse, poisoning, adolescents, cannabis, new psychoactive substances, public health

## Abstract

This six-year multicentre study investigated acute intentional poisoning with substances of abuse in adolescents to identify changes and patterns in substance use. Data from 562 adolescents were collected from three paediatric poison centres in Romania between January 2017 and December 2022. This study analysed the epidemiological and sociodemographic characteristics of the adolescents, including age, gender, place of residence, history of substance abuse, psychiatric history, and history of institutionalised care. The findings revealed that cannabis and new psychoactive substances (NPSs) are the most commonly implicated substances, each with distinct profiles among adolescents. Cannabis was involved in 46.1% of cases, with a significant association with urban residency. NPSs were identified as the second most prevalent substance, accounting for 39.3% of cases. These were more prevalent in rural areas and among patients with psychiatric disorders. Cannabis and NPSs were also the most commonly implicated substances in acute intentional poisoning cases with substances of abuse. These substances have distinct profiles among adolescents, including age, gender, residency area, history of substance abuse, psychiatric history, and institutional care. These findings underscore the necessity of targeted public health interventions and integrated care approaches to address substance use and related mental health issues in adolescents.

## 1. Introduction

The global problem of substance abuse poses a serious threat to public health and child safety, with significant consequences extending into adolescence. Any involvement in substance abuse at an early age can disrupt normal developmental processes and profoundly affect a child’s future trajectory. Early exposure to substance abuse has been consistently associated with long-term physical, behavioural, social, and health risks [1,2,3,4]. It is common for the majority of individuals who engage in substance abuse during adulthood to have initiated its use during their adolescence [5].

Adolescence is a critical period characterised by vulnerability to substance use, which can lead to significant consequences such as the development of problematic substance use patterns, substance use disorders, and other psychiatric conditions such as depression, anxiety, and conduct disorder. In addition, substance use during this formative period can alter the brain development in the regions responsible for higher-order cognitive functions and inhibitory control, posing long-term cognitive challenges [6].

Adolescents make up 20% of the world’s population and are particularly at risk of substance abuse [6]. Among 15–24-year-olds, about 18.2% (8.6 million) reported using cannabis in the last year, with 9.6% (4.5 million) using it in the last month [7]. Cannabis remains a significant problem, being the second most commonly reported substance in acute drug toxicity presentations in the European Drug Emergencies Network (Euro-DEN) Plus hospital network in 2021; it was involved in 25% of such cases, often in combination with other substances [7].

According to the European Union Drugs Agency (EUDA), previously named the European Monitoring Centre for Drugs and Drug Addiction, among individuals aged 15–24, an estimated 18.6% (8.8 million) used cannabis in 2023. In the 15–34 age group, approximately 2.0% (2.0 million) are daily or almost daily cannabis users. Trends in cannabis use at the national level are mixed; of the countries that conducted surveys since 2021 and reported confidence intervals, three reported higher usage estimates, eight remained stable, and two reported a decrease compared to previous surveys [8].

In recent decades, new psychoactive substances (NPSs) have become a growing concern. By the end of 2023, the EUDA was monitoring around 930 new psychoactive substances, of which 41 were reported in Europe for the first time in 2022. National estimates of last-year NPS use among young adults (aged 15–34) vary widely, from 0.1% in Latvia to 5.1% in Romania [8].

The prevalence of other illicit drugs is also striking. In the European Union, surveys suggest that almost 2.5 million young adults (aged 15–34) used cocaine and 1.5 million used amphetamines in 2023. In addition, 2.2 million young adults have reported using MDMA during the same period [8].

Specifically in Romania, data from the Romanian Observatory of Drugs and Drug Addiction show that among 15–34-year-olds, rates of lifetime use, last-year use, and last-month use of any illicit drug are higher than those in the general population, at 16.9%, 10%, and 6.6%, respectively [9]. There has been a significant increase in last-month use, with a 1.4-fold increase compared with previous surveys [9]. Among 16-year-old students, lifetime use of any illicit drug is 9.5%, with 9% reporting use in the past year [8]. Notably, among those who have used cannabis in the last 12 months, more than one-quarter have also used ecstasy (29.3%) and cocaine (26.3%), and a significant proportion have also used amphetamines (20.7%), methamphetamines, or new psychoactive substances (around 16%) [9].

National data regarding Romanian adolescents who use substances of abuse or seek medical assistance for substance abuse poisoning are currently lacking. The Romanian Observatory for Drugs and Drug Addiction reports the data regarding drug usage among adolescents in conjunction with that of young adults, making it difficult to devise separate preventive strategies for younger individuals. Additionally, no studies have been found by the authors that examine the attributes of patients to recognise patterns and trends.

This six-year multicentre study aims to describe patterns and emerging trends in acute intentional poisoning that occur during recreational use of substances of abuse among adolescents, providing critical insights into the evolving landscape of adolescent substance use and its implications for public health strategies.

## 2. Materials and Methods

### 2.1. Study Design

This study employed an observational retrospective cohort analysis of data from three paediatric poison centres.

This study included patients under the age of 18 who were admitted to the hospital for acute intentional poisoning with substances of abuse between 1 January 2017 and 31 December 2022. Patients with acute intentional poisoning resulting from suicide attempts were not included in this study. This study adhered to the ethical principles outlined in the Declaration of Helsinki and was approved by the Ethics Committee of the hospital (Approval No. 11358, dated 18 March 2024). All guardians of the patients included provided informed consent for their participation.

This research analysed the epidemiological and sociodemographic characteristics of adolescents who had self-poisoned with substances of abuse.

### 2.2. Data Collection

Data were extracted from electronic health records using the International Classification of Diseases, 10th Revision, Australian Modification (ICD-10-AM) codes T40.0–T40.9 (narcotics and psychodysleptics), T42.3–T42.8 (antiepileptics, sedative–hypnotics, and antiparkinsonism drugs), and T43.0–T43.9 (psychotropic drugs), in addition to external cause codes X61–X64 (intentional self-poisoning). The variables collected included date of admission, age, sex, residential area, care in an institutional setting, psychiatric history, substance abuse history, circumstances of the poisoning event, and involved substances.

### 2.3. Definitions

The implicated substances were determined upon hospitalisation by considering the substances reported by the patient and the findings of a clinical examination performed by a senior consultant in clinical toxicology [10,11].

### 2.4. Statistical Analysis

Statistical analyses were performed using XL-STAT version 2023.5 (Addinsoft, Paris, France) and VassarStats version SCR-010263 (Vassar College, New York, NY, USA). Continuous variables were expressed as means with standard deviations (SDs), while categorical data were presented as frequencies and percentages. Yates correction of the chi-square test and standardised residuals were used to assess the relationships between categorical variables [12]. One-way ANOVA was conducted to compare the median age values across different subgroups. The temporal trends of frequencies were assessed using the Cochran–Armitage test [13,14]. A *p*-value of <0.05 was considered statistically significant.

## 3. Results

### 3.1. Patient Characteristics and Poisoning Circumstances

The cohort comprised 562 adolescents who presented with acute intentional poisoning with substances of abuse during the study period.

**Figure 1:**
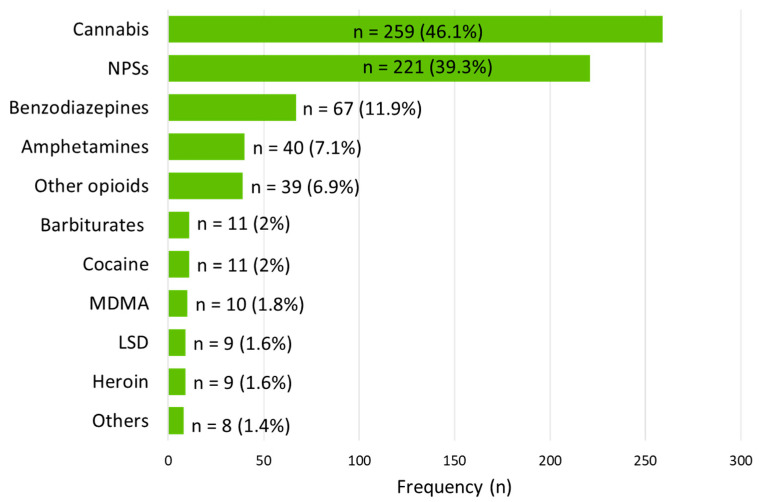
The frequency of substances of abuse involved in acute intentional poisoning cases. Abbreviations: NPSs, new psychoactive substances; MDMA, methylenedioxymethamphetamine; LSD, lysergic acid diethylamide.

The substances identified as the primary cause of poisoning were cannabis (46.1%, n = 259), NPSs (39.3%, n = 221), benzodiazepines (11.9%, n = 67), amphetamines (7.1%, n = 40), opioids (8.5%, n = 48), and other substances in 2% or less each (Figure 1). The acute poisoning cases involved a single substance of abuse in 445 cases (79.2%), two substances in 74 cases (13.2%), and three or more substances in 43 cases (7.7%). According to all cases where adolescents were poisoned by more than one substance, 84 out of 117 cases (71.8%) involved the concurrent use of cannabis and other drugs. The most common combinations were cannabis with benzodiazepines in 31 cases, followed by cannabis with amphetamines in 17 cases, and cannabis with opioids in 15 cases.

The acute poisoning events occurred in 37 (6.6%) cases in the patient’s home, 31 (5.5%) cases in someone else’s residence, and 127 (22.6%) cases in public places. In 65.3% of cases (n = 367), the location of the poisoning was not specified.

**Figure 2:**
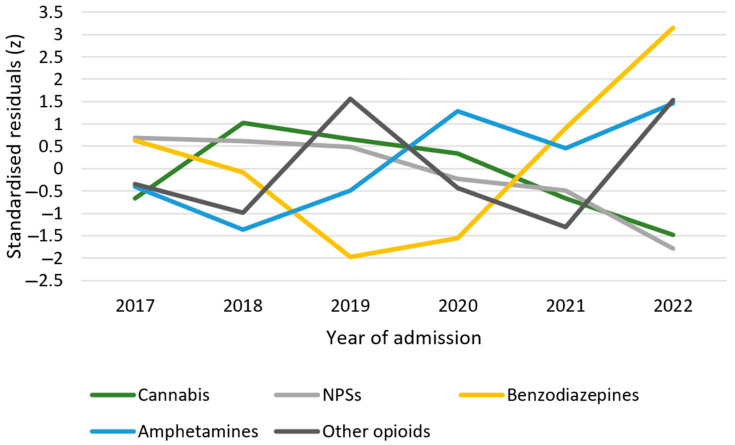
The temporal trends of the substances of abuse involved in acute intentional poisoning cases. Abbreviations: NPSs, new psychoactive substances.

A decrease in annual admissions was noted (R^2^ = 0.44), but the proportion of these patients among all acute poisoning cases remained consistent throughout the study period. The frequency of the involved substances remained constant throughout the study period (Figure 2), except for benzodiazepines, which showed higher-than-expected incidences in 2022 (z = 2.53), and amphetamine poisoning, which exhibited a trend of increasing incidence over time (χ^2^(1) = 4.45; *p* = 0.03).

The age distribution of the patients ranged from 11 to 18 years, with a mean age of 15.56 years (SD = 1.37) for the overall cohort. The ages of the patients significantly varied depending on the involved substance (*p* = 0.01), demonstrating that those who had heroin poisoning were younger, while those with amphetamine or cocaine poisoning were older. Notably, concurrent ethanol use, along with substance abuse, was significantly more common in female patients (*z* = 3.51; χ^2^ = 25.27; *p* < 0.001).

**Figure 3:**
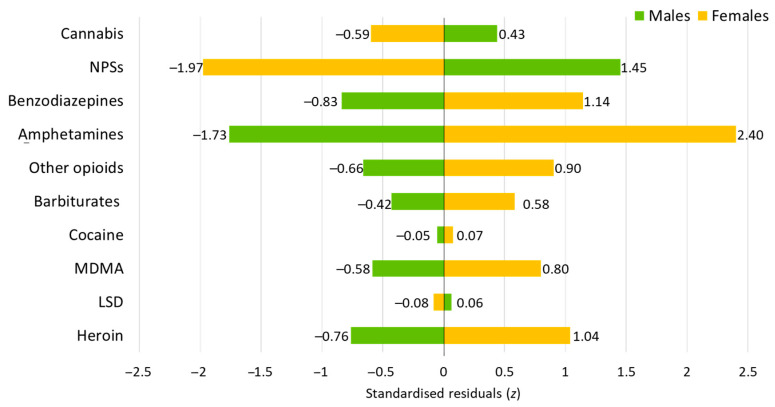
The distribution of patient sex by the substances of abuse involved in acute intentional poisoning cases. Abbreviations: NPSs, new psychoactive substances; MDMA, methylenedioxymethamphetamine; LSD, lysergic acid diethylamide.

The overall cohort comprised a predominantly male population (65.3%; n = 367), reflecting a 2:1 male-to-female ratio. Nevertheless, there were notable variations in sex distribution among the substances involved (Figure 3). For instance, NPS poisoning demonstrated a higher prevalence in males (z = 1.45), while benzodiazepines and amphetamines showed a higher prevalence in females (z = 1.14 and z = 2.40, respectively). An analysis of age by sex showed that female patients had a significantly lower mean age (15.28 years old; SD = 1.36) than their male counterparts (15.69 years old; SD = 1.36; *p* < 0.001).

**Figure 4:**
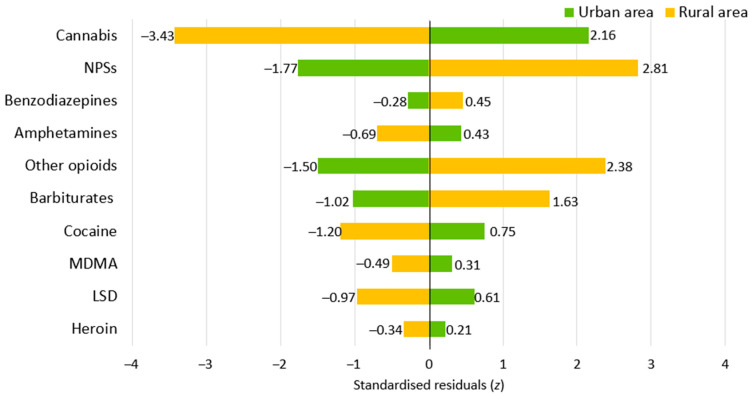
The distribution of patient area of residence by the substances of abuse involved in acute intentional poisoning cases. Abbreviations: NPSs, new psychoactive substances; MDMA, methylenedioxymethamphetamine; LSD, lysergic acid diethylamide.

Patients were predominantly from urban areas (69.6%; n = 391). However, the distribution of residence areas varied significantly depending on the incriminated substances (Figure 4). For instance, individuals who had been poisoned with cannabis (z = 2.16) were predominantly from urban areas, whereas those who had been poisoned with NPSs (z = 2.82), opioids (z = 2.39), or barbiturates (z = 1.63) were predominantly from rural areas.

**Figure 5:**
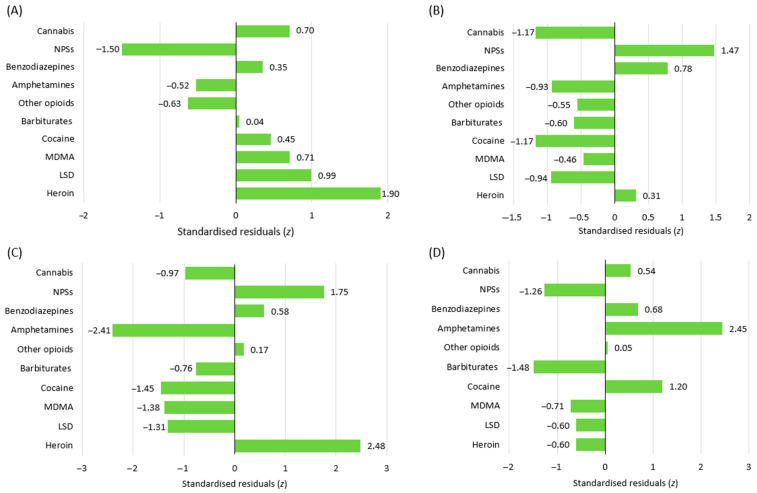
(**A**–**D**) The distribution of chronic substance abusers (**A**), patients with psychiatric disorders (**B**), patients receiving care in an institutional setting (**C**), and the co-ingestion of ethanol beverages (**D**) by the substances of abuse involved in acute intentional poisoning cases. Abbreviations: NPSs, new psychoactive substances; MDMA, methylenedioxymethamphetamine; LSD, lysergic acid diethylamide.

Chronic substance abuse was identified in 280 cases, accounting for 49.8% of the total. Moreover, this issue was more frequently associated with heroin poisoning (z = 1.90) and less frequently with NPS poisoning (z = −1.5; see Figure 5A). Out of all patients, 152 (27.0%) had documented psychiatric disorders, which were more frequently associated with NPS poisoning (z = 1.47; see Figure 5B). The presence of institutional care was observed in 114 (20.3%) patients, with this issue being more frequent in cases involving NPSs (z = 1.75) and heroin (z = 2.48), and less frequent in cases of amphetamine poisoning (z = −2.41; see Figure 5C). Chronic substance use was significantly linked to both institutional care (z = 2.71; *p* < 0.001) and the presence of psychiatric disorders (z = 4.49; *p* < 0.001).

Ethanol co-ingestion was documented in 113 patients (20.1%) and was more frequently associated with amphetamine poisoning (z = 2.45, see Figure 5D).

The substances of abuse most frequently incriminated in cases of acute poisoning were cannabis and NPSs. The results for patients poisoned with one of these substances are detailed in the next two subsections.

### 3.2. Cannabis Poisoning

Patients presenting with cannabis poisoning had a mean age of 15.66 years (SD = 1.27), in accordance with the overall cohort. The sex distribution in the cannabis poisoning subgroup was 2:1 male-to-female, which aligned with the sex distribution observed in the overall cohort (Figure 3). In addition, there was a predominance of cases from urban areas (z = 2.16; Figure 4) when analysing the distribution of patients by area of residence.

A temporal analysis revealed a constant distribution of cannabis poisoning cases over the study period, with no significant trend (χ^2^(5) = 8.66, *p* = 0.12). However, in 2022, the frequency of cannabis poisoning was lower than the expected distribution for this particular year (z = −1.48; Figure 2).

The observed occurrence of cannabis poisoning in institutionalised patients, chronic drug users, and the alcohol co-exposure group was consistent with the distribution of cases in the study population. By contrast, the observed frequency of cannabis poisoning cases among patients with a documented psychiatric history was lower than expected based on the overall distribution of cases in our cohort (z = −1.17; Figure 5B).

### 3.3. NPS Poisoning

An analysis of NPS poisoning cases among adolescents revealed a mean age of 15.39 years (SD = 1.42), reflecting the age distribution of the cohort. The male-to-female ratio in NPS poisoning cases was 2.7:1, indicating a higher prevalence in males than females (z = 1.45, Figure 3). Furthermore, patients from rural areas were over-represented, with a frequency that was higher than expected (z = 2.81; Figure 4).

The annual occurrence of NPS poisoning remained consistent throughout the study period (χ^2^(5) = 6.99; *p* = 0.22). However, a significant trend towards a decrease in frequency over time was observed (χ^2^(1) = 5.39; *p* = 0.02). This trend peaked in 2022, when the lowest number of cases was recorded (z = −1.78; see Figure 2).

NPS poisoning was observed more often than expected in institutionalised patients (z = 1.75; Figure 5C) and in those with a documented psychiatric history (z = 1.47; Figure 5B). By contrast, it occurred less frequently than expected among chronic abusers (z = −1.50; Figure 5A). Furthermore, co-exposure to ethanol alongside NPS poisoning occurred less commonly than anticipated (z = −1.26; Figure 5D).

## 4. Discussion

### 4.1. Patient Characteristics and Poisoning Circumstances

This study included 562 adolescents with a gender distribution in line with the literature and similar to the one observed in adult populations [15,16,17,18]. The distribution by area of residency and the mean age of the patients were similar to previous findings as well [19,20]. Female patients presented at a significantly younger age than males, suggesting different behavioural patterns or social pressures influencing substance use initiation between these sexes. This consistent gender disparity in age at presentation has been documented in the literature [20,21]. Chronic substance use was significantly associated with institutional care and psychiatric disorders, highlighting the vulnerability of youth in institutionalised settings and those with mental health issues. A significant proportion of the cohort had documented psychiatric disorders, a co-occurrence well documented in the existing literature, underscoring the need for integrated care addressing both substance use and mental health [15,22,23]. Additionally, 20% of patients received institutional care, reinforcing the link between substance use and institutional care [16]. These findings emphasise the need for targeted interventions for institutionalised youth and those with psychiatric disorders.

Cannabis was the most involved substance in acute poisoning cases, followed by new psychoactive substances, benzodiazepines, and opioids, aligning with the most recent report of the EUDA [8]. The Romanian Observatory for Drugs and Drug Addiction’s 2023 national report similarly highlighted NPSs and cannabis as the most used substances, and the American National Center for Drug Abuse Statistics’ 2023 report noted cannabis as the most used substance, followed by opioids and LSD [9,24,25]. The high prevalence of cannabis and NPSs in our cohort is consistent with broader epidemiological trends showing an increasing use of these substances among adolescents [26]. Age variations in substance use may indicate differences in accessibility, social acceptability, and risk perception, necessitating targeted prevention strategies tailored to specific adolescent groups.

Most poisoning incidents involved a single substance, but 13.2% involved two substances, and 7.7% involved three or more, indicating significant polysubstance use risk. Ethanol co-ingestion occurred in 20.1% of cases, with a gender difference as follows: females were more likely to consume ethanol with other substances, suggesting different usage patterns influenced by social or behavioural factors. No significant link was found between ethanol co-ingestion and residence area, indicating widespread behaviour across environments. The clinical impact of alcohol co-ingestion along with other substances is well documented in the literature, though gender distribution data are scarce [27,28,29,30,31].

Despite a decrease in admissions per year, the proportion of acute poisoning cases among all admissions remained stable, suggesting persistent underlying issues. The lack of significant monthly admission trends suggests that acute poisoning is a constant concern rather than seasonal. However, seasonality has been noted in substance abuse-related deaths in England and Wales and in adolescent alcohol and drug use in the U.S. [32,33].

The majority of the poisoning incidents occurred in unknown locations, with many happening in public places, complicating monitoring and prevention efforts. Although the literature on the location of substance abuse incidents is limited, a 2020 Canadian paediatric hospital study reported that cannabis poisonings often occur in private residences [34].

### 4.2. Cannabis Poisoning

The present study provides a comprehensive analysis of cannabis poisoning among adolescents, examining demographic and geographic patterns, temporal trends, and associated characteristics. The mean age of the patients was 15.66 years, reflecting the overall cohort and the literature, highlighting mid-adolescents’ vulnerability to cannabis-related harm during crucial developmental stages [35,36]. The sex distribution within the cannabis poisoning subgroup showed a 2:1 male-to-female ratio, consistent with the overall cohort and broader trends in adolescent substance use, where males are more prone to risk-taking behaviours [37,38,39,40]. Understanding these gender differences is crucial for developing targeted prevention and intervention strategies. Another noteworthy finding was that cannabis poisoning cases were more prevalent in urban areas, aligning with Canadian studies reporting higher rates in urban centres due to the greater availability of cannabis products in cities [41,42]. Unexpectedly, cannabis poisoning frequency among patients with documented psychiatric histories was lower than expected, contrasting with studies reporting higher substance use among those with psychiatric disorders [43,44].

No significant temporal trend in cannabis poisonings was identified, except for a slight decrease in 2022. This contrasts with North America, where cannabis poisoning has increased post-legalisation, especially with edible cannabis products mistaken for food by children [45,46]. In Romania, where cannabis remains illegal, the stable incidence may be due to strict enforcement and limited availability, unlike in Canada and some U.S. states, where legalisation has increased access and poisoning incidents, highlighting the impact of legal frameworks on substance use patterns. The slight decrease in 2022 may indicate effective law enforcement, public health campaigns, or shifts in youth behaviour during the COVID-19 pandemic [47,48]. Conversely, North America’s post-legalisation increase shows the complexities of drug policy changes, where increased availability and perceived safety lead to higher use and accidental poisonings, particularly with edibles [49].

### 4.3. NPS Poisoning

The results for NPSs revealed important trends. A mean age of 15.39 years and a male-to-female ratio of 2.7:1 indicate a higher NPS poisoning prevalence among males, consistent with global patterns [8,26,50]. Our finding that NPS poisoning is more common in rural areas contrasts with trends typically observed for other substances, which often show higher incidences in urban areas. This rural predominance could be due to several factors, including differences in availability, socioeconomic dynamics, or reporting practices in rural versus urban areas.

Internationally, the NPS landscape has evolved, with the EUDA noting fewer new substances in the last years, likely owing to tighter regulations [8]. Similarly, the United Nations Office on Drugs and Crime has reported that 2022 saw the lowest number of new NPSs reported in over a decade. This reduction could indicate effective international and national efforts to control the proliferation of these substances [50]. Our study showed a significant decrease in NPS poisoning cases over time, with the lowest number of cases recorded in 2022. This decline aligns with international trends, indicating that stricter regulations are effective. The most recent European Drug Report highlights legislative measures reducing new psychoactive substances entering the market [8].

The high occurrence of NPS poisonings among institutionalised patients and those with psychiatric histories in our study highlights their vulnerability, consistent with other regions where mental health issues increase substance abuse risk [50]. By contrast, lower NPS poisoning rates among chronic abusers and those co-exposed to ethanol suggest different usage patterns or substance preferences.

### 4.4. Benzodiazepine Poisoning

The analysis of acute benzodiazepine poisonings in Romanian adolescents revealed a mean age of 15.63 years, with a higher prevalence among females (male-to-female ratio of 1.3:1). Over the study period, the frequency of benzodiazepine poisonings showed significant variability, with a notable increase in cases in 2022.

This trend mirrors global patterns, with rising misuse and poisonings involving benzodiazepines and, especially, opioids. The CDC notes increased overdoses involving illicit benzodiazepines like etizolam and flualprazolam [51,52]. In Europe, the EUDA also reports concerns about benzodiazepine misuse, especially with other substances [8,53]. The trend of increasing benzodiazepine-related poisonings mirrors patterns observed in Romania, with fluctuations in prevalence likely influenced by changes in drug availability and public health measures. The increased benzodiazepine poisonings among females in our study align with broader observations that young females more often misuse prescription medications, linked to higher anxiety and depression rates, underscoring the need for targeted interventions [51]. The spike in benzodiazepine poisonings in 2022 may reflect the COVID-19 pandemic’s impact. This period saw rising mental health issues and medication misuse. Increased benzodiazepine prescriptions during the pandemic support this, as individuals sought to manage stress and anxiety, reflecting a broader trend of heightened medication use [54,55]. Furthermore, an editorial in *Frontiers in Psychiatry* discusses how the COVID-19 pandemic led to increased benzodiazepine use, supported by a prospective observational study in Catalonia that recorded higher benzodiazepine prescriptions from March 2020 to December 2021 compared with the previous two years [56]. This increase is indicative of a global trend where the pandemic exacerbated mental health issues, leading to greater reliance on medications for relief.

### 4.5. Amphetamine Poisoning

The analysis of acute amphetamine poisoning among adolescents revealed a mean age of 16 years and a higher prevalence among females (male-to-female ratio of 0.7:1). This contrasts with U.S. data but aligns with increasing use among European females [8,57]. Globally, trends in amphetamine use and poisoning have been inconsistent. U.S. data show rising amphetamine-related emergency visits due to both illicit and prescription misuse, such as with Adderall and Ritalin [58]. The results also revealed a consistent incidence of amphetamine poisoning over the study period, peaking in 2020, reflecting increased pandemic-related stress and drug use. The increased stress, social isolation, and changes in drug availability during the pandemic contributed to increased amphetamine use and poisoning. The National Institute on Drug Abuse (NIDA) reported that drug overdoses, including those involving amphetamines, increased during the pandemic owing to factors such as increased stress and decreased access to treatment [59]. Amphetamine poisoning was less common among institutionalised patients but often involved co-exposure to ethanol. The significant association with co-exposure to ethanol is consistent with other research indicating that polysubstance use, particularly with alcohol, is common among amphetamine users [60].

### 4.6. Opioids Poisoning

The findings regarding acute opioid poisonings provide valuable insights into the demographic and geographic patterns of these incidents. The mean age of patients with acute intentional heroin poisoning was 14.78 years, which is slightly lower than the overall cohort’s mean age but consistent with international data [61,62]. Heroin poisoning was more prevalent among individuals in institutional care, with chronic substance abuse, and in rural areas, mirroring the U.S. literature [63,64]. No significant trend was observed in the prevalence of heroin poisoning over the study period. The consistent prevalence of opioid poisonings over the study period, without a significant upward or downward trend, contrasts with the escalating opioid crisis observed in North America [61,62,65,66]. This stability could be attributable to the implementation of effective regulatory measures, differences in drug availability, or variations in reporting practices between regions.

### 4.7. Barbiturate Poisoning

The analysis of barbiturate poisonings revealed several key findings. Barbiturate poisonings were more frequent among patients from rural areas and less common in those with co-exposure to ethanol. The temporal trends exhibited variability, with peaks in 2017 and 2018 and fewer cases in subsequent years.

Globally, barbiturate use has declined due to the availability of safer alternatives like benzodiazepines. However, barbiturate toxicity remains significant, especially in intentional overdoses and suicides. Australian data indicate declining hospitalisations but rising deaths from barbiturate misuse [67].

### 4.8. Cocaine Poisoning

The mean age of patients with acute intentional cocaine poisoning in the study was 16.45 years; this was slightly higher than the overall cohort. This finding aligns with data from the United States, in which adolescent cocaine use is commonly observed in older adolescents [68]. The low incidence in rural areas and institutionalised patients suggests that environmental factors influence cocaine use. Co-exposure to ethanol is common, increasing health risks [69]. The unusual spike in cocaine poisonings in 2021 may align with disruptions caused by the COVID-19 pandemic, which impacted drug availability and use patterns across various regions [70].

### 4.9. MDMA Poisoning

The patterns of MDMA poisoning exhibited annual fluctuations, peaking in 2020. This indicates variability in MDMA use or reporting within this population. Notably, the occurrence of MDMA poisoning was lower among patients in institutional care. A lower incidence in institutional care suggests variability in use or reporting, but further research is needed regarding this matter. Globally, MDMA remains prevalent in urban nightlife areas. The European Drug Report 2023 and U.S. data indicate fluctuating MDMA use and poisonings [8,71].

### 4.10. LSD Poisoning

LSD poisoning in adolescents showed a low incidence, with one or two cases annually, consistent with the literature [72]. According to national surveys in Europe, the estimated prevalence of use of LSD and other hallucinogens among young adults aged 15–34 in 2023 is 1% or less [8]. A particularly noteworthy finding in our study is the lower-than-expected prevalence of LSD poisoning among patients in institutional care. The literature on the correlation between LSD poisoning and institutional care settings is limited, indicating an area for further research.

### 4.11. Poisoning with Other Substances

Less common substances were involved in eight cases of poisoning identified in our study. Four cases involved glue inhalation, specifically “prenandez”, highlighting concerns about its accessibility and neurotoxic effects [73]. The other four cases involved ingesting toxic plant parts (*Datura stramonium* and *Atropa belladonna*). Both plants contain potent psychoactive alkaloids that can cause severe neuropsychiatric and cardiotoxic effects. These findings are consistent with reports in the literature that emphasise the dangers of plant-based intoxications, especially in regions where such plants are readily accessible [74].

### 4.12. Strengths and Limitations

This study’s strengths include its multicentre design, including data from multiple paediatric poison centres, which provided a comprehensive overview of adolescent poisoning incidents. The six-year study duration allowed for a detailed examination of temporal trends and substance use patterns among adolescents. This study is notable for its valuable epidemiological insights into associations between chronic substance use, type of care, psychiatric disorders, substances involved in poisonings, and socio-clinical factors. It addresses the gaps in the literature on this age group, shedding light on the relationships between adolescent substance abuse and clinical and social determinants.

One of the limitations is that our study includes data from the main three paediatric poison centres, which cover almost all regions of Romania, but could potentially limit the applicability of the findings to other populations or regions. Additionally, focusing on adolescents treated at paediatric poison centres might introduce selection bias by excluding less severe cases managed elsewhere. Therefore, the sample used in this study is deemed representative of adolescents in Romania who have been poisoned by substances of abuse, but it may not accurately represent the broader population of Romanian adolescents who use substances of abuse.

Data completeness and accuracy challenges in retrospective designs may lead to inconsistencies. The observational nature introduces the possibility of inadequately addressing confounding variables such as socioeconomic status, family dynamics, and medical histories. Please note that, due to the retrospective nature of this study and the need for cost-effective management of patients in the emergency department, analytical toxicological analyses were not routinely conducted to confirm the suspected substances. Consequently, it is possible that the diagnosis may not be entirely accurate in certain instances.

## 5. Conclusions

Poisoning with substances of abuse remains a persistent public health concern, with a steady number of cases reported annually. This study highlights that cannabis and NPSs are among the most frequently involved substances in acute intentional poisoning incidents. The demographic and clinical profiles of adolescents affected by these substances vary significantly. Cannabis poisoning is more prevalent in urban areas and less frequently associated with a psychiatric history. Conversely, NPS poisoning is more common in rural areas and among patients in institutional care, and it is significantly associated with a psychiatric history. 

Developing tailored prevention and intervention strategies that are specific to the unique risk profiles associated with each substance is of paramount importance. In order to enhance the effectiveness of prevention programmes and target high-risk groups, it is essential for professionals working in the field of drug abuse prevention to have a thorough understanding of the primary categories of adolescents who use substances of abuse and the main locations where these substances are used. Our study emphasises these key findings, which can be utilised to improve community-based and school-based programmes as well as to develop new programmes that are focused on patients with psychiatric disorders and those in institutional care.

## Data Availability

The data that support the findings of this study are available from the corresponding author upon reasonable request due to privacy concerns.
